# Cohort profile: systemic lupus erythematosus in Sweden: the Swedish Lupus Linkage (SLINK) cohort

**DOI:** 10.1136/bmjopen-2015-008259

**Published:** 2015-08-14

**Authors:** Elizabeth V Arkema, Julia F Simard

**Affiliations:** 1 Clinical Epidemiology Unit, Department of Medicine, Karolinska Institute, Stockholm, Sweden; 2 Division of Epidemiology, Department of Health Research and Policy, Stanford School of Medicine, Stanford, California, USA; 3 Division of Immunology and Rheumatology, Department of Medicine, Stanford School of Medicine, Stanford, California, USA

**Keywords:** EPIDEMIOLOGY, IMMUNOLOGY, RHEUMATOLOGY

## Abstract

**Purpose:**

A cohort of individuals with systemic lupus erythematosus (SLE) was identified through linkage of several national registers to investigate important epidemiological questions using not only population-based data to minimise selection bias, but also to identify matched comparators from the general population to serve as controls. This cohort was established to overcome the general dearth of data in SLE epidemiology.

**Participants:**

All individuals registered in Sweden with a personal identity number and who have obtained medical care at any hospital or public non-primary outpatient specialist care with suspected SLE were identified. Inpatient register data date back to the 1960s, although complete national coverage of the inpatient register was achieved in 1987. In 2001, the outpatient component was also added to the register, representing the entire country of Sweden. For each suspected individual with SLE, up to five individuals from the general population were identified and matched on sex, birth year and county of residence.

**Findings to date:**

We have linked this study population to a number of national and quality registers in Sweden to identify first-degree relatives, deaths, births, dispensed prescriptions, comorbidities and disease end points, such as stroke and cancer, as well as basic health economic data. We found geographic variability in the prevalence of SLE by county. We have also shown that being first-born confers a reduced odds of having SLE in childhood and early adulthood.

**Future plans:**

In addition to updating the national register linkage with several more years of follow-up data, we are adding several quality registers in Sweden, including the Tuberculosis register and the Social Insurance Office database. While these updates are ongoing and additional follow-up accumulates, we are studying a number of outcomes in SLE, including stroke, pregnancy and death. We will continue to present findings at scientific conferences and in the peer-reviewed literature.

**Strengths and limitations of this study**

- The SLINK cohort was created using prospectively collected, population-based data minimizing selection and recall biases.
- Information on a large number of exposures, outcomes and covariates are included.
- Date of onset, disease activity measures and serology are unavailable, which may limit the identification of incident SLE or SLE phenotypes.

## Introduction

Systemic lupus erythematosus (SLE) is a rheumatic autoimmune disease characterised by chronic inflammation and autoantibodies with manifestations ranging from fevers, arthritis and rashes to renal failure and neurological disease. SLE is heterogeneous and poorly understood, both in terms of its diagnosis and management. Females are more likely to be diagnosed with SLE, and the female predominance is most pronounced during their childbearing years. However children, postmenopausal women, and men of all ages are at risk of SLE. Worldwide prevalence ranges from 3 to over 200 per 100 000 people and varies by age, sex, race/ethnicity and region.^1^

Large, population-based SLE cohorts are rare but important to advancing our understanding of the disease aetiology, trajectory and outcomes. The Swedish Lupus Linkage (SLINK) cohort was set up to facilitate epidemiological research in SLE by improving power and providing extensive longitudinal follow-up data in a relatively unselected population-based study.

The establishment of a population-based cohort focused on SLE not only addresses the overwhelming dearth of data in SLE research, but also uniquely incorporates data on individuals from the general population. Many epidemiological studies in SLE are conducted in clinical samples or are secondary to larger initiatives such as the Nurses’ Health Studies. The matched cohort design allows multiple controls from the general population to be sampled for each case, which allows investigators to identify population risks and baseline rates of events of interest.

## Cohort description

The study was designed by investigators at the Clinical Epidemiology Unit at the Karolinska Institute in Sweden and funded by the Strategic Program in Epidemiology's Young Scholars in Epidemiology Award at the Karolinska Institute. To date, SLINK has been used in a handful of studies: to assess the prevalence of SLE in Sweden on 1 January 2010; to study the use of register data in SLE case identification; to examine perinatal risk factors for SLE; and to describe reproductive outcomes among women with SLE.

Using methods previously established in register-based studies in Sweden, we conducted a national register linkage using nearly one dozen national registers. There is no formal recruitment for the register-identified population of patients. Individuals were included in the cohort as possible SLE ‘cases’ (SLE exposed) on the basis of their routine clinical care. Sweden provides its residents and citizens with universal health coverage through their personal identity number (*personnummer*), which acts as a medical record number, social security number and identifier for most social services. A small percentage of individuals opt to purchase and use private insurance. Although this number may be growing, individuals seeking their care *exclusively* in the private setting using private insurance may be missed.

Individuals were identified from the National Patient Register as possible SLE cases if they were discharged with an SLE diagnosis (main or contributory) using International Classification of Disease (ICD) coding. At each medical care interaction, physicians specify at least one main diagnosis and multiple contributory diagnoses for both inpatient care and specialist outpatient visits. These diagnosis codes were searched to identify the pool of possible SLE cases in the entire Swedish population. Using these two data sources, inpatient and outpatient data, approximately 16 000 individuals with *possible SLE* were identified between 1964 and 2013, with at least one discharge diagnosis for SLE. When restricting to at least two SLE coded visits in the Patient Register, ignoring same day visits, we identified 11 205 individuals. Defining SLE with at least two ICD-coded visits is preferable and consistent with what others have done to reduce misclassification and increase comparability. A formal validation study is currently underway.

**Figure 1:**
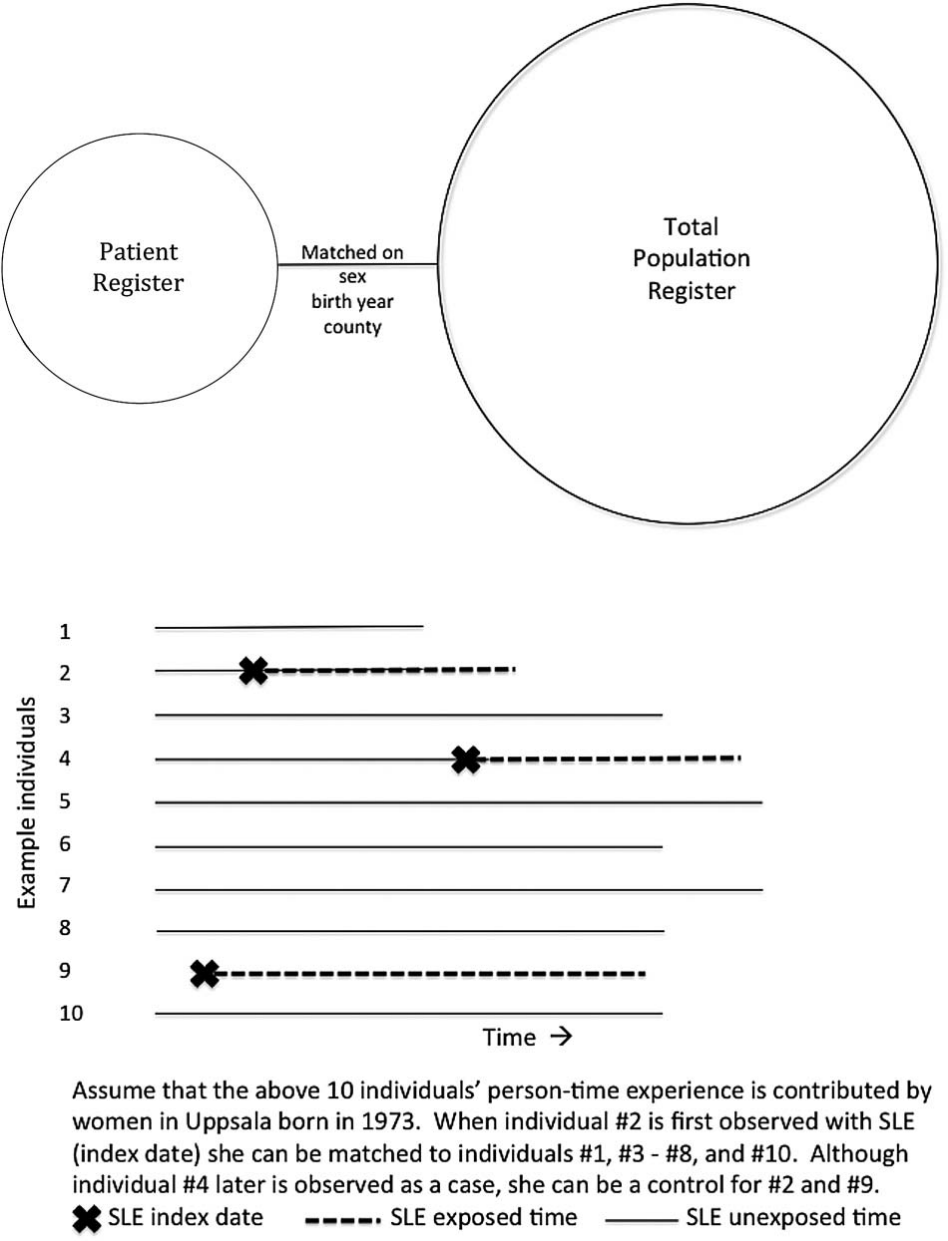
Study population schematic diagram for national Swedish register linkage from the first iteration. The updated linkage uses the Patient Register exclusively to identify systemic lupus erythematosus (SLE) cases.

To be included in the study population, individuals were labelled as possible SLE cases based on the diagnosis of a single inpatient or outpatient visit, with the understanding that to reduce misclassification, validated algorithms will be used to define SLE cases in future research. Each possible SLE case was matched to five individuals randomly selected from the general population accounting for birth year, sex and county of residence. Similar to incidence density sampling, each general population comparator was restricted to be ‘free of disease’ (ie, have no SLE ICD codes in the Patient Register) before the date of the first observed SLE ICD code (index date) of their matched case (figure 1). As with similar sampling schemes in case–control studies, this does not preclude the general population comparators from developing and being diagnosed with SLE in the future. We are able to identify when this occurs using the longitudinal Patient Register data. The study population was then linked to the Multi-Generation Register to identify first-degree relatives of both the SLE and comparator populations.

Subsets of patients with SLE can be further identified based on requiring additional SLE-coded visits, prescription dispensation from the Prescribed Drug Register and SLE-coded discharge diagnosis from a clinic or department most likely to see patients with SLE (rheumatology, nephrology, dermatology, internal medicine and paediatrics). Using multiple definitions not only allows for more direct comparison with other studies, but also may reduce misclassification by removing false positives from the potential case population.

### Characteristics of the study population

The study population was identified using decades of healthcare utilisation data from the National Patient Register: individuals with suspected SLE and a general population comparator from 1971 to the end of 2013. Register data (see below) are available both before and after the index dates for every individual in the study population. The available population-based data are routinely collected by agencies such as the National Board of Health and Welfare, the Social Insurance Office and Statistics Sweden. Individuals who leave the system can be identified via census and migration data from Statistics Sweden or identified in the Cause of Death register.

The current study population has complete follow-up up to at least 31 December 2013. The entire study population has been (or soon will be for those marked with an asterisk (*)) linked to the registers described below prospectively and retrospectively, when possible. Approval for this entire linkage has been provided by the local ethics board at the Karolinska Institute. A number of protective measures were taken to maintain anonymity, including removing all identifying information and replacing unique personal identity numbers with randomly generated unique identifiers.

- *National Patient Register* began in 1964 and includes information on all inpatient admissions in specific counties. In 1987, all Swedish counties were included. In 2001, the register was expanded to include data from non-primary outpatient care throughout Sweden. For both inpatient and outpatient components, data on admission and discharge dates, clinic, county, operating codes (where relevant) and discharge diagnoses are included using the calendar-year-specific ICD codes. Data on comorbidities treated in inpatient or outpatient care, and surgeries are available both retrospectively and prospectively. A recent study confirmed that on average 85–95% of diagnoses in the inpatient register were valid.^2^
- The *Prescribed Drug Register (PDR)* began in July 2005 and includes all pharmacy dispensations to the patient at any of the national pharmacies (all Swedish pharmacies are included). Data include name and anatomical therapeutic code (ATC) for any dispensed medication, prescribed/dispensed dose, date and total cost.
- The *Cancer Register* began in 1958 and includes nearly all cancer cases in Sweden since its start. Reporting is mandatory and nearly 100% complete. In addition to date of diagnosis and type of cancer, information is available on tumour-node-metastasis-staging and pathology.
- In 2004, a separate *Basal Cell Cancer Register* was started as this subset of skin cancers was not routinely reported before.
- The *Cause of Death Register* contains all deaths in Sweden, including date, primary cause of death and up to 20 contributory underlying causes of death. Data are collected from the death certificates.
- *Longitudinal Integration Database of Health Insurance and Labour Market Studies (LISA)* provides data on highest level of education and unemployment benefits such as sick leave and disability pension.
- The *Medical Birth Register* includes data on all births in Sweden from 1973 onwards, including maternal health, medications, smoking, pregnancy history, labour and delivery characteristics, and fetal outcomes. Data include information and history from the first antenatal visit through birth/delivery. Initially, only pregnancies ≥28 weeks were included in the register. In 2008, pregnancies ≥22 weeks were included. Some data, such as cigarette smoking and snus use, were added in the early 1980s.
- The Swedish Stroke Register (Riks-Stroke)* includes all acute stroke incidents in hospitals since 1998 (with some limited data from 1994 onwards). Information is available on risk factors, diagnosis, follow-up, complications, drug therapy and ABCD^2^ score (a clinical prediction rule based on five parameters: age, blood pressure, clinical features, comorbid diabetes and duration of transient ischaemic attack (TIA) used to estimate the risk of stroke after a TIA).
- The *Total Population Register* has census-related information on individuals, including migrations and county of residence on 31 December of each year.
- The Tuberculosis (TB) Register* contains data on active TB diagnoses from all of Sweden. In Sweden, it is mandatory for clinicians to report to the TB register's web-based reporting system any individual they start on treatment for TB. In the same system, any positive finding of *Mycobacterium tuberculosis* is also reported and linked to the clinician's report by the patient's unique personal identity number. Quality and completeness of the data retrieved is monitored weekly, resulting in 100% coverage of all cases verified by culture.
- Svevac* provides data on immunisations since late 2002, including timing, administration, type and care unit that administered or facilitated the vaccination. In 2006, Svevac began registering human papillomavirus vaccination, which is complemented by the PDR to account for subsidised vaccinations delivered from the pharmacy system.
- The Social Insurance Office (Försäkringskassan)* complements the LISA register by providing data on the exact dates of sick leave and disability pension, along with the extent of this benefit.

## Findings to date

We recently reported the 2010 prevalence of SLE in Sweden and found that the prevalence varied by age and sex, as expected. Register-based prevalence ranged from 85 per 100 000 people for the least strict definition (one ICD coded visit) to 46 per 100 000 for the most strict definition (at least two ICD coded visits, at least one ICD code from a specialist and at least one dispensing of a prescription used in SLE). We also detected geographic variability that was not explained by population differences nor healthcare facility or provider differences.^3^

Utilising the linkage to the Medical Birth Register, we investigated perinatal risk factors for future SLE and observed an association between birth order and odds of incident SLE.^4^ Previously observed associations with preterm birth and birth weight in adult women from the Nurses’ Health Studies were not confirmed.^5^ We are currently examining maternal and fetal outcomes of pregnancy in SLE and have confirmed previous reports of increased risks of numerous pregnancy and fetal complications when looking at first singleton pregnancy in SLE mothers and mothers from the general population.^6^

## Strengths and limitations

The Swedish universal healthcare system includes all citizens and residents with a personal identity number and covers the large majority of the population. This sizeable cohort is derived from prospectively collected, population-based data minimising both selection and recall biases. Some analyses, particularly stratified analyses or subsets of cases defined by SLE phenotypes, may be limited by low power. We cannot exclude the possibility of misclassification. Our initial classification and validation work show varying degrees of misclassification associated with register-based definitions in these data, but suggest that including at least a single visit coded with an SLE ICD code from an outpatient clinic to define SLE (as opposed to relying on hospitalisation data only) has excellent properties (high sensitivity, specificity, and positive and negative predictive values). The SLINK cohort and the goal of this work is not to identify all SLE in Sweden to serve as a national register, but instead to use the already existing registers to identify a large cohort of patients for this epidemiological study to overcome longstanding limitations of data and power.

Using these data to study onset or risk factors for incident disease may be a limitation. SLE typically has long induction and latent periods, limiting the identification of aetiologically relevant exposure windows or true incidence of disease. The earliest date of observation in the registers may not represent the symptom onset nor diagnosis date. Therefore, identification of potential risk factors should be carried out thoughtfully. Furthermore, clinical information, such as disease activity measures, autoantibodies and serology, are not available in these data.

This cohort provides longitudinal follow-up and retrospective data on a myriad of exposures, outcomes and other covariates of interest. However, some potential confounders or effect modifiers may not be available for the entire study population or for different study periods. For example, some smoking information is available in the Medical Birth Register, but not in the Patient Register.

## Collaboration

We invite those hoping to collaborate to contact Dr Simard to determine whether specific scientific questions can be addressed in these data. Owing to the nature of these data, they are to remain de-identified and in Sweden. Analysis of these data will need to be conducted within the Karolinska system for the time being. However, we do not think that this obstacle should discourage interested parties from collaborating.

## References

[1] Danchenko N, Satia JA, Anthony MS Epidemiology of systemic lupus erythematosus: a comparison of worldwide disease burden. Lupus 2006;15:308–18. 10.1191/0961203306lu2305xx 16761508

[2] Ludvigsson JF, Andersson E, Ekbom A External review and validation of the Swedish national inpatient register. BMC Public Health 2011;11:450 10.1186/1471-2458-11-450 21658213 PMC3142234

[3] Simard JF, Sjowall C, Ronnblom L Systemic lupus erythematosus prevalence in Sweden in 2010: what do national registers say? Arthritis Care Res (Hoboken) 2014;66:1710–17. 10.1002/acr.22355 24757083

[4] Arkema EV, Simard JF Perinatal risk factors for future SLE: a population-based nested case-control study. Lupus 2015;24:869–74. 10.1177/0961203315570160 25672372

[5] Simard JF, Karlson EW, Costenbader KH Perinatal factors and adult-onset lupus. Arthritis Rheum 2008;59:1155–61. 10.1002/art.23930 18668600 PMC2748190

[6] Arkema EV, Palmsten K, Sjowall C Pregnancy outcomes in SLE: before and after. Arthritis Rheumatol 2014;66 (S10):S826.

